# Gender equality and women’s health at the workplace: where we stand?

**DOI:** 10.1192/j.eurpsy.2026.10273

**Published:** 2026-07-31

**Authors:** T. Kurimay

**Affiliations:** Psychiatry and Psychatric Rehabilitation, Teaching Dep. of Semmelweis University, North-buda Saint John Central Hospital Family Centred Mental Health Centre, Budapest, Hungary

## Abstract

**Abstract:**

Gender equality in the workplace is increasingly recognised as a key determinant of mental health, yet it is still often framed as a structural or policy issue rather than a core clinical concern. In psychiatry, where work-related stress, role overload and social expectations directly shape symptom presentation, diagnostic pathways and treatment outcomes, this separation is particularly problematic. Early career psychiatrists—both residents and young consultants—occupy a unique position: they deliver frontline clinical care while simultaneously navigating hierarchical, gendered work environments themselves.

This talk provides a concise, evidence-informed overview of where we currently stand regarding gender equality and women’s health at work, with a focus on its relevance for everyday psychiatric practice. Drawing on epidemiological data, occupational mental health research and clinical experience, it highlights how gendered workplace exposures—such as unequal caregiving burdens, reduced autonomy, discrimination and “invisible work”—translate into well-established psychiatric risk factors, including chronic stress, burnout, depression and anxiety.

The presentation moves beyond theory to focus on practical implications. It explores common gender-related biases in assessment and diagnosis, the importance of routinely integrating women’s health and life-course factors into psychiatric evaluations, and the clinical value of validation-focused communication. In addition, it addresses the workplace as a determinant of mental health for both patients and psychiatrists, linking concepts such as double standards, token roles and leaky pipelines to early career vulnerability and professional sustainability.

Finally, the talk offers concrete, low-threshold strategies that early career psychiatrists can apply immediately in clinical encounters and team settings—micro-level actions that promote equity without requiring formal leadership roles. The central message is that championing gender equality at work is not an ideological stance, but a component of clinically accurate, ethically sound and sustainable psychiatric practice.

**Disclosure of Interest:**

None Declared

